# Signatures of resolution reprogramming in the 5xFAD mouse model

**DOI:** 10.1002/alz70855_105663

**Published:** 2025-12-24

**Authors:** Eric D Hamlett, Galina Kondrikova, Dariusz Pytel

**Affiliations:** ^1^ Medical University of South Carolina, Charleston, SC, USA

## Abstract

**Background:**

Specialized pro‐resolving mediators (SPMs) promote inflammatory resolution and homeostasis and are thought to have specific reprogramming effects on human microglia. Decreased SPM levels have been correlated with chronic neuroinflammation, late‐stage Alzheimer's disease (AD) and neuropathology in humans, yet few studies have explored the cellular signatures of resolution. Amyloid is though to bind one target resolution receptor, ChemR23, leading to internalization. In this study we explore if chronic administration of the specific ChemR23‐targeted SPM, Resolvin E1 (RvE1), alters memory performance and inflammation 5xFAD transgenic mice at 12 months of age when pathology burden and memory impairment is substantial. We hypothesize the RvE1 may have significant recuse potential.

**Method:**

At 11 months of age we administered, RvE1 (10 per kg body weight/day), for 1.5 months with subcutaneous mini‐osmotic pumps. A 9‐day sequential series of memory performance was administered before and after treatment to quantify novel object recognition, hyperactivity, spatial context memory, and cognitive resilience. We quantified cytokine responses in blood and brain tissue using standard immuno‐dot blots, multiplexed ELISA and quantitative RNA assay. We measured RvE1 and other lipids from brain and other organs to confirm pharmacokinetics. We examined the occurrence and degree of plaque and microglial morphology in multiple brain regions using immunofluorescence microscopy.

**Result:**

RvE1 significantly reduces microglial activation, cytokine expression and plaque burden in amyloid challenged 5xFAd mice. We discovered that RvE1 leads to compensatory response in specific ChemR23 receptor and other resolution‐oriented G protein coupled receptor family. We are performing single cell analysis to better ascertain how RvE1 altered cellular resolution response in different classes of cells.

**Conclusion:**

We have demonstrated that RvE1 modulates inflammation in a mouse model and in a microglial cell line. Resolution pathways may represent drugable targets to reduce neuroinflammation associated with neuropathology, but a more thorough understanding of how RvE1 achieves therapeutic benefits is needed.

